# Soluble and insoluble dipeptide repeat protein measurements in *C9orf72*-frontotemporal dementia brains show regional differential solubility and correlation of poly-GR with clinical severity

**DOI:** 10.1186/s40478-020-01036-y

**Published:** 2020-11-09

**Authors:** Annelies Quaegebeur, Idoia Glaria, Tammaryn Lashley, Adrian M. Isaacs

**Affiliations:** 1 National Hospital for Neurology and Neurosurgery, Queen Square, London, WC1N 3BG UK; 2 UK Dementia Research Institute at UCL, Cruciform Building, Gower Street, London, WC1E 6BT UK; 3 Department of Neurodegenerative Disease, UCL Queen Square Institute of Neurology, London, UK; 4 Research Support Service, Institute of Agrobiotechnology, CSIC-Government of Navarra, Mutilva, Spain; 5 Queen Square Brain Bank for Neurological Studies, Department of Clinical and Movement Neurosciences, UCL Queen Square Institute of Neurology, London, UK

**Keywords:** Frontotemporal dementia, Frontotemporal lobar degeneration, *C9orf72* mutation, Dipeptide repeat proteins, Poly-GR, Solubility, Meso Scale Discovery

## Abstract

A *C9orf72* repeat expansion is the most common genetic cause of frontotemporal dementia (FTD) and amyotrophic lateral sclerosis. One of the suggested pathomechanisms is toxicity from dipeptide repeat proteins (DPRs), which are generated via unconventional translation of sense and antisense repeat transcripts with poly-GA, poly-GP and poly-GR being the most abundant dipeptide proteins. Animal and cellular studies highlight a neurotoxic role of poly-GR and poly-PR and to a lesser degree of poly-GA. Human post-mortem studies in contrast have been much less clear on a potential role of DPR toxicity but have largely focused on immunohistochemical methods to detect aggregated DPR inclusions. This study uses protein fractionation and sensitive immunoassays to quantify not only insoluble but also soluble poly-GA, poly-GP and poly-GR concentrations in brain homogenates of FTD patients with *C9orf72* mutation across four brain regions. We show that soluble DPRs are less abundant in clinically affected areas (i.e. frontal and temporal cortices). In contrast, the cerebellum not only shows the largest DPR load but also the highest relative DPR solubility. Finally, poly-GR levels and poly-GP solubility correlate with clinical severity. These findings provide the first cross-comparison of soluble and insoluble forms of all sense DPRs and shed light on the distribution and role of soluble DPRs in the etiopathogenesis of human *C9orf72*-FTD.

**Electronic supplementary material:**

The online version of this article (10.1186/s40478-020-01036-y) contains supplementary material, which is available to authorized users.

## Introduction

Frontotemporal dementia (FTD) and amyotrophic lateral sclerosis (ALS) are considered to be part of a disease spectrum most often caused by a non-coding hexanucleotide GGGGCC repeat expansion in the *C9orf72* gene [10, 14, 24]. How these repeat expansion mutations mediate neurotoxicity in C9-FTD/ALS disease is a hotly debated topic in the field. Three main mechanisms have been put forward: loss of C9orf72 function and gain of function via toxicity from repeat RNAs and/or their dipeptide repeat protein products [3]. Sense and antisense expanded repeat transcripts undergo unconventional repeat-associated non-AUG dependent (RAN) translation generating five different proteins of repeating dipeptides (poly-GA, poly-GP, poly-GR, poly-PR and poly-PA) referred to as dipeptide repeat proteins (DPRs) [2, 12, 22, 23]. These DPRs accumulate in aggregated form, which in post-mortem brains of *C9orf72*-mutant FTD and/or ALS patients are visible on immunohistochemistry as p62-positive, predominantly neuronal cytoplasmic inclusions [1, 27].

Recent work suggests that loss- and gain-of-function mechanisms play a synergistic role in causing neurodegeneration in experimental in vivo and in vitro studies [4, 35]. With new therapeutic strategies on their way, it is of great importance to determine the relative contribution of the various pathomechanisms in human disease as well. Research over the last decade has however yielded conflicting results so far regarding the role of the different DPRs in C9-FTD/ALS. DPR-mediated neurotoxicity has been extensively demonstrated in overexpression studies in experimental animal and cellular model studies. The arginine-rich poly-GR and poly-PR in particular have been shown to be highly toxic and are implicated in disrupting several cellular processes known to be disturbed in C9-FTD/ALS disease such as nucleocytoplasmic transport and RNA processing [3, 30, 34]. In contrast, the majority of human post-mortem studies have generally failed to show a consistent link between DPR inclusions and clinical severity or neurodegeneration [8, 9, 16, 18, 19, 28]. Nevertheless, two recent studies showed a relationship between poly-GR inclusions and neurodegeneration [25, 26] with additional evidence that methylated poly-GR correlates with clinical severity [15].

An important limitation is that DPR research in human post-mortem tissue has largely relied on immunohistochemical analyses of DPR inclusions to quantify DPR load, and therefore has not addressed a possible contribution of soluble DPRs. Indeed, the concept of soluble oligomers rather than the insoluble protein aggregates and inclusions acting as drivers of the neurotoxicity is an emerging theme in several other neurodegenerative disorders such as Alzheimer’s and Parkinson’s disease [6]. Also, in C9-FTD/ALS there is evidence that the existence of a soluble form of DPRs precedes the aggregation into an insoluble aggregate [13, 18], however its potential role in mediating toxicity in C9-FTD/ALS remains to be determined. A single study has reported on soluble and insoluble fractions of poly-GP and poly-GA [13], but a role for the possibly more toxic soluble poly-GR has not been addressed so far. Therefore our aim was to quantify and compare soluble and insoluble DPRs, including poly-GR, in brain regions with various degrees of clinical involvement in C9-FTD.

## Materials and methods

### Case and tissue selection

The brains used in this study were donated to the Queen Square Brain Bank for Neurological Disorders and tissue is stored for research under a license issued by the Human Tissue Authority (No. 12198). This study has been approved by the NHS Health Research Authority East of England—Essex Research Ethics Committee. All selected cases (n = 13) had a clinical syndrome in keeping with frontotemporal dementia (FTD), a confirmed hexanucleotide repeat expansion mutation in *C9orf72* and showed FTLD-TDP type A pathology on neuropathological examination. Two cases also developed additional signs of motor neuron disease (FTD-MND). Clinical details on age of onset, age at death and post-mortem interval were available for all cases (Table 1). Fresh frozen tissue was taken from the anterior frontal cortex (middle frontal gyrus), temporal cortex (middle temporal gyrus), occipital cortex (pericalcarine cortex) and cerebellum (cerebellar cortex) with the former two representing areas that are clinically affected in FTD and the latter two representing areas that are clinically minimally affected. Five brains of neurologically healthy subjects were used as control cases. The clinical, pathological and genetic data of the FTD and control cases are detailed in Table 1 and Additional file 1: Table 1 respectively.

**Table 1:** Demographical details of *C9orf72*-FTD subjects

Number	Gender	Pathology	Genetics	Age of onset (years)	Age at death (years)	Disease duration (years)	Post-mortem interval (h:min)
1	M	FTLD-TDP type A	*C9orf72*	64	73	9	61:08
2	F	FTLD-TDP type A	*C9orf72*	58	66	8	107:05
3	M	FTLD-TDP type A	*C9orf72*	59	65	6	30:00
4	M	FTLD-TDP type A	*C9orf72*	66	71	5	51:52
5	F	FTLD-TDP type A	*C9orf72*	57	62	5	63:05
6	M	FTLD-TDP type A	*C9orf72 (homozygous)*	43	45	2	25:53
7	M	FTLD-TDP type A	*C9orf72*	54	60	6	32:20
8	F	FTLD-TDP type A	*C9orf72*	56	67	11	85:35
9	M	FTLD-TDP type A	*C9orf72*	52	58	6	49:45
10	M	FTLD-TDP type A	*C9orf72*	53	63	10	77:20
11	M	FTLD-TDP type A	*C9orf72*	62	68	6	99:00
12	F	FTLD-TDP type A	*GRN/C9orf72*	58	66	8	115:00
13	F	FTLD-TDP type A	*C9orf72*	66	74	8	85:50

### Brain tissue homogenization

Sequential extractions of the brain tissue were performed as follows. Briefly, approximately 150 mg of frontal, temporal, occipital and cerebellar cortex was lysed in ice-cold RIPA buffer (Sigma-Aldrich) supplemented with protease inhibitor (Roche cOmplete mini EDTA-free) to make a 10% homogenate and subsequently disrupted on ice using a handheld rotor–stator homogenizer (Tissue Ruptor II, Qiagen). After a low speed clearance spin, 2,000 g for 4 min, protein concentration was determined by *DC* Protein Assay (BioRad). Lysates were centrifuged at 100,000 g for 30 min at 4 °C to obtain “soluble” fractions. The resulting pellets were extracted in fresh 7 M urea, sonicated and cleared at 100,000 g for 30 min at 22 °C. These “insoluble” fractions were brought to 1 M urea by adding TBS with protease inhibitors.

### Meso Scale Discovery immunoassay

Meso Scale Discovery (MSD) electrochemiluminescence detection technology was utilised to establish sandwich immunoassays to detect poly-GR, poly-GP and poly-GA dipeptide repeats. Previously described rabbit anti-(GR)_7.5_ antibody [29], newly generated affinity purified rabbit anti-(GP)_8_ antibody (Eurogentec) and a commercially available mouse monoclonal anti-(GA) antibody (MABN889 Millipore) were used as capture, coating with 2, 2 and 1 µg/ml respectively. Detection on a MSD sector imager was performed with biotinylated versions of the same antibodies, loading 1, 2 and 2 µg/ml respectively, followed by sulfo-tagged streptavidin. Specificity was confirmed with a cross-reactivity assay using lysates from HeLa cells transfected to express different C9orf72 RAN translated proteins [(GR)_100_, (GA)_100_, (PR)_100_ and (GGGGCC)_92_] (Additional file 1: Figure 1).

The volume loaded per well for each assay were made to correspond to 180 µg of soluble fractions and 4.5 times that amount for insoluble fractions. Volumes were adjusted to load 90 µl of RIPA or 1 M of urea solution. Freshly extracted samples were run in duplicate, avoiding freeze–thaw. The median of intra-plate coefficient of variation (CV) between duplicates was 2.7% for poly-GR, 2.3% for poly-GP and 2.4% for poly-GA. HeLa transfected lysates were run on all plates to assess inter-plate assay variability. The assays were performed in two batches: 3 plates were run per day over 2 days, yielding inter-plate CV values of 6.5%, 4.9% and 19.1% for poly-GR, poly-GP and poly-GA assays. Three weeks later a second batch of 12 plates were run over 4 days, giving intra-plate CVs of 14.6%, 10.7% and 18.6% for poly-GR, poly-GP and poly-GA assays respectively. Inter-plate variability across the two batches was higher (31.8% for poly-GR, 23.8% poly-GP and 51.1% poly-GA). Control cases and calibrator standards were run on every plate to help control for inter-plate variability. MSD Discovery Workbench software that subtracts the value from a blank calibrator from each reading to correct for background was used to interpolate concentrations against a four-parameter logistic regression curve fitted to the values obtained loading serial dilutions of calibrators. (GR)_7.5_, (GP)_8_ synthetic peptides or GST-(GA)_36_ recombinant protein were utilised as calibrators in each assay plate diluted either in RIPA or 1 M urea with protease inhibitors. Average concentrations of control cases were subtracted from samples. The researchers performing the brain tissue homogenization and MSD immunoassay were blinded to the condition.

### Statistical analysis

For comparison of DPR concentrations and ratios between different brain areas, data analysis was undertaken with Friedman test (non-parametric, paired ANOVA) with Dunn’s correction for multiple comparisons. Differences between the DPR concentrations relative to values in control cases were assessed via unpaired *T* test with Welch’s correction. Ratios of soluble versus insoluble fractions were compared via Wilcoxon matched-pairs signed ranked test. Correlative analyses were performed with Spearman’s correlation test. No additional correction for multiple comparisons was performed. GraphPad Prism version 8.0 for Mac was used for all data analyses and graphing. In all analyses a *p*-value of less than 0.05 was considered statistically significant.

## Results

### Patient demographics

Soluble and insoluble fractions of dipeptide repeat proteins (DPRs) poly-GP, poly-GA and poly-GR were measured in post-mortem brain from 13 subjects carrying a hexanucleotide repeat expansion mutation in the *C9orf72* gene. All patients had shown symptoms of frontotemporal dementia (FTD) and post-mortem neuropathology examination in all showed FTLD-TDP type A pathology. One of the patients (patient 12, [17]) had a *progranulin* (*GRN*) mutation in addition to a *C9orf72* mutation and a single patient showed a homozygous mutation for *C9orf72* (patient 6, [11]). Five post-mortem brains from neurologically healthy subjects were used as a control group. The demographical details and the post-mortem interval of the C9-FTD and control groups are listed in Table 1 and Additional file 1: Table 1, respectively. There was no significant difference in post-mortem interval between the C9-FTD and control groups (C9-FTD 68.0 ± 8.2 h versus healthy control 79.4 ± 8.5 h, *p* = NS).

### Soluble and insoluble poly-GA, poly-GP and poly-GR can be specifically detected in C9-FTD brains

**Fig. 1:**
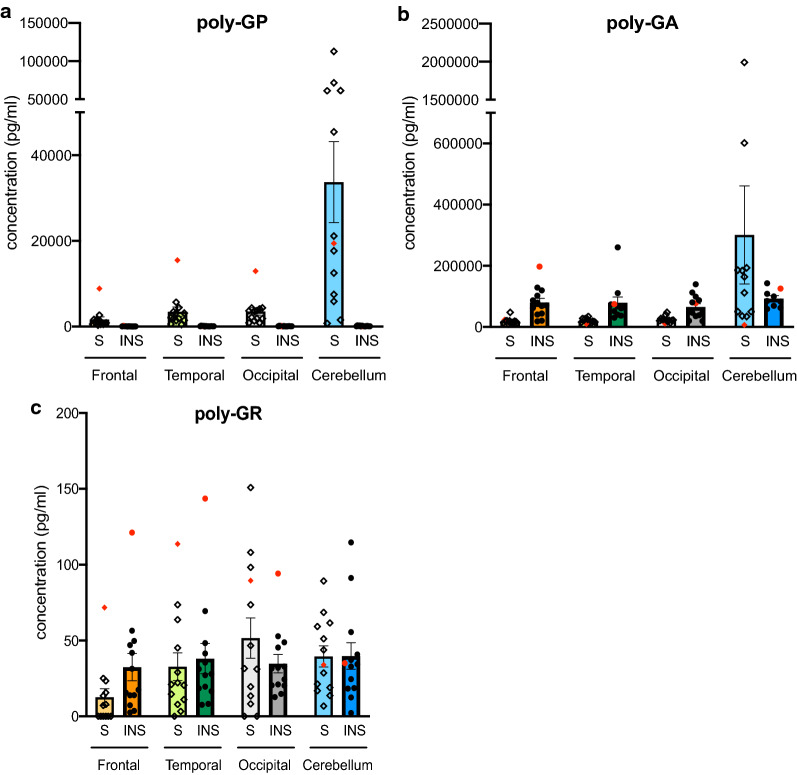
Comparison of soluble (S) and insoluble (INS) DPR levels. Poly-GP (**a**), poly-GA (**b**) and poly-GR (**c**) concentrations in frontal cortex (Frontal), temporal cortex (Temporal), occipital cortex (Occipital) and cerebellum (Cerebellum). For each area, soluble fractions are shown as the left bar with individual data points represented as diamonds (◇) and insoluble fractions are shown as the right bar with individual data points illustrated as circles (•). All data are mean ± SEM. For statistical analyses see Figs. 2 and 3. The homozygous *C9orf72* repeat expansion case is shown in red

Soluble and insoluble fractions of DPR proteins in different neuroanatomical regions were measured by MSD immunoassay. The brain areas were selected to reflect different degrees of neurodegeneration and clinical involvement in C9-FTD with frontal and temporal cortex chosen as most affected areas and occipital and cerebellar cortex as less involved regions. Concentrations of soluble and insoluble poly-GA and poly-GP protein vastly exceeded background values (Table 2, Additional file 1: Figure 2) and poly-GA showed higher soluble and insoluble concentrations than soluble and insoluble poly-GP respectively in all examined brain areas (Fig. 1). In contrast, poly-GR levels were much closer to, yet statistically different from the values obtained in the control brains (Fig. 1, Table 2, Additional file 1: Figure 2). In the single case carrying a homozygous *C9orf72* repeat expansion mutation the concentrations of all measured DPRs were generally considerably higher than the average concentrations however not in the cerebellum (Additional file 1: Table 2).

**Table 2:** DPR levels are significantly higher in *C9orf72*-FTD patients than healthy control subjects

	Control	C9orf72-FTD	*p*-value
GP soluble	1.0 ± 0.07	248 ± 70	0.008
GP insoluble	1.0 ± 0.06	13 ± 1.1	< 0.0001
GA soluble	1.0 ± 0.44	28 ± 13	0.039
GA insoluble	1.0 ± 0.28	31 ± 2.7	< 0.0001
GR soluble	1.0 ± 0.08	1.4 ± 0.09	0.004
GR insoluble	1.0 ± 0.12	3.5 ± 0.31	< 0.0001

The data represent the relative DPR values in the *C9orf72*-FTD subjects as measured by MSD in comparison to the values measured in the healthy control subjects in frontal, temporal, occipital cortex and cerebellum. Data are shown as mean ± SEM. Right column shows *p*-values. Statistical analysis: unpaired t-test with Welsch correction

### Soluble DPR levels are less abundant in clinically affected areas

**Fig. 2:**
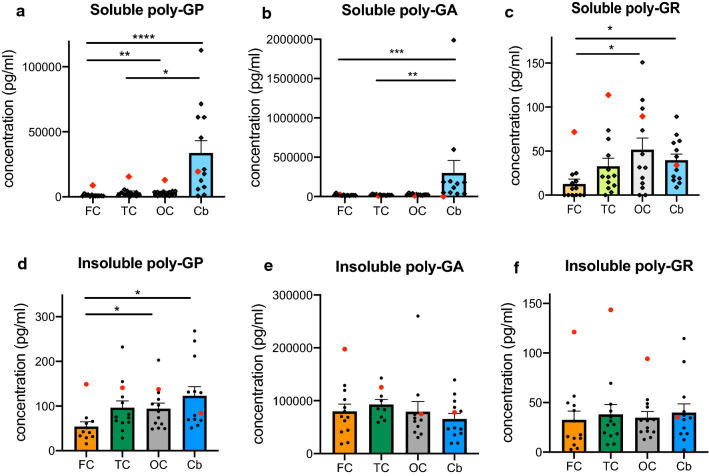
DPR protein concentrations across different brain regions. Soluble (**a**–**c**) and insoluble (**d**–**f**) poly-GP, poly-GA and poly-GR levels are depicted across frontal cortex (FC), temporal cortex (TC), occipital cortex (OC) and cerebellum (Cb). The homozygous *C9orf72* mutation case is shown in red. All data are mean ± SEM. Statistical analysis: Friedman test followed by Dunn’s multiple comparison test. *p < 0.05, **p < 0.01, ***p < 0.001, ****p < 0.0001

Next we analysed the distribution of both insoluble, and the less examined, soluble DPR proteins across brain regions with variable degrees of clinical involvement (Fig. 2). Soluble poly-GP, poly-GA and poly-GR levels were significantly higher in the cerebellum than in the frontal cortex (poly-GP in frontal cortex 1639 ± 629 pg/ml versus cerebellum 33719 ± 9458 pg/ml, *p* < 0.0001; poly-GA in frontal cortex 19,468 ± 2878 versus cerebellum 299,371 ± 160,068 pg/ml, *p* < 0.001; poly-GR in frontal cortex 12.7 ± 5.5 pg/ml versus cerebellum 39.7 ± 6.9 pg/ml, *p* < 0.05). Soluble poly-GP and poly-GA in the cerebellum were also higher than levels in the temporal cortex, and occipital cortex levels of soluble poly-GP and poly-GR were higher than levels in the frontal cortex. These findings indicate that soluble DPR levels are generally less abundant in the clinically more affected areas i.e. frontal and temporal cortex.

Assessment of insoluble DPRs revealed that insoluble poly-GP was higher in the cerebellum and occipital cortex when compared to frontal cortex (Fig. 2d), which is similar to soluble poly-GP, yet to a lesser degree. In contrast, insoluble poly-GA and poly-GR did not show significant differences across the examined brain regions (Fig. 2e–f), indicating that insoluble poly-GA and poly-GR show different anatomical distributions in comparison to their soluble counterparts.

### DPR solubility profiles are both DPR- and region-specific

**Fig. 3:**
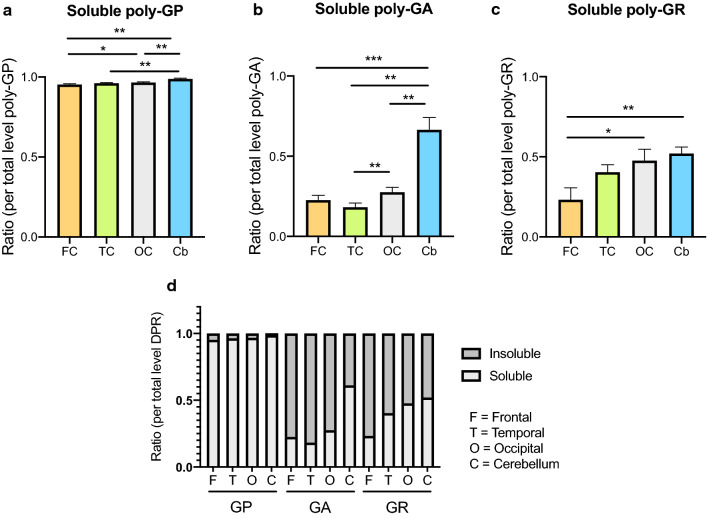
Regional solubility of DPR proteins. The ratio of soluble poly-GP (**a**), poly-GA (**b**) and poly-GR (**c**) are compared between frontal cortex (FC), temporal cortex (TC), occipital cortex (OC) and cerebellum (Cb). All data are mean ± SEM. Statistical analysis: Friedman test followed by Dunn’s multiple comparison test. *p < 0.05, **p < 0.01, ***p < 0.001. (**d**) Ratios of soluble (S) and insoluble (INS) DPR per total level of DPR in frontal cortex (F), temporal cortex (T), occipital cortex (O) and cerebellum (C). The results of the statistical analysis comparing soluble ratios in between brain regions are shown in (**a**–**c**)

Comparing soluble versus insoluble levels revealed striking differences in solubility profiles between the various DPR proteins (Fig. 3, Additional file 1: Figure 3). The vast majority of poly-GP was present as a soluble form (soluble fraction ranging from 96 to 99% across the various regions, Fig. 3a, d, Additional file 1: Figure 3a–d). This is in major contrast to poly-GA, which was predominantly present as insoluble protein (insoluble fraction ranging from 72 to 82% except for cerebellum, see next paragraph, Fig. 3b, d, Additional file 1: Figure 3e–h). Poly-GR showed a more equal distribution between soluble and insoluble states (insoluble fraction ranging from 47 to 76%, Fig. 3c, d, Additional file 1: Figure 3i–l).

In addition to a different solubility dependent on the type of DPR protein, there was also a region-specific solubility profile with the cerebellum showing the highest soluble protein proportions. The change in solubility was most striking for poly-GA: whereas in the cerebral cortical regions it was mainly insoluble, the soluble protein constituted 61% of the total poly-GA in the cerebellum (Additional file 1: Figure 3e–h, Fig. 3d). In line with this, the ratio of soluble DPR protein levels (over total DPR protein) in the cerebellum was significantly higher in comparison to the soluble fraction in the frontal cortex for all DPR proteins (relative increase of soluble DPR in cerebellum versus frontal cortex: 2.8 for poly-GA, 2.2 for poly-GR and 1.03 for poly-GP; *p* < 0.05; Fig. 3a–c). Also in comparison to the ratio of soluble DPR load in the temporal cortex, higher values were noted in the cerebellum for poly-GA and poly-GP (Fig. 3a–b).

### Correlation of DPR levels with clinical parameters

Overall there was remarkable variability in DPR protein levels between individual C9-FTD brains. Importantly, there was no obvious influence of post-mortem delay on any of the DPR protein fractions (Additional file 1: Table 3), indicating that protein degradation due to longer post-mortem interval did not contribute to this variability.

**Fig. 4:**
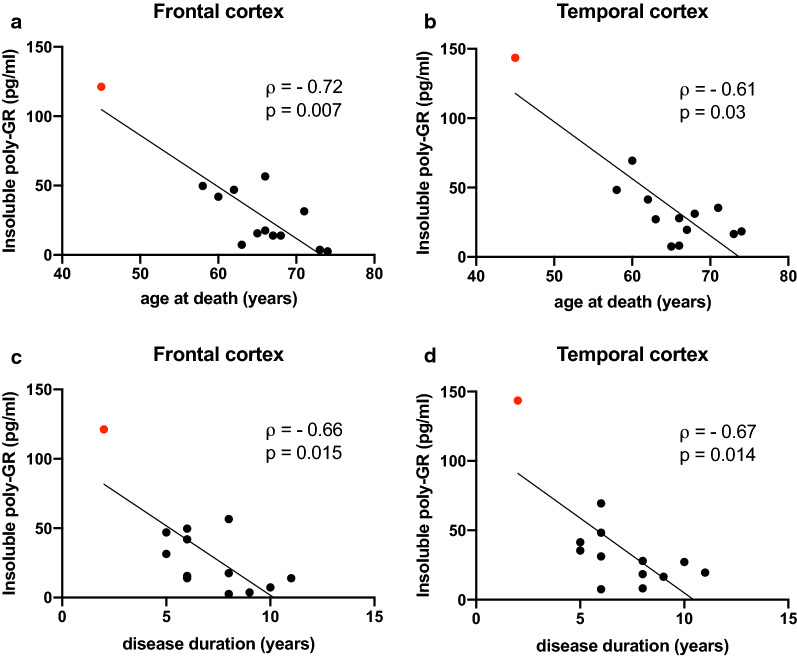
Insoluble poly-GR levels correlate with clinical parameters. Correlation of insoluble poly-GR levels in frontal and temporal cortex with age at death (**a**–**b**) and disease duration (**c**–**d**). The data points in the graphs represent individual *C9orf72* cases (with homozygous *C9orf72* mutation case in red). Spearman’s correlation analysis performed with Spearman’s correlation coefficient ρ. All data are shown in Table 3

We next addressed the question whether DPR protein concentrations were associated with age of onset, age at death and/or disease duration. Interestingly, insoluble poly-GR in the clinically affected frontal and temporal cortex showed a negative correlation with age at death and disease duration (Fig. 4, Table 3). A trend of correlation was also noted between insoluble poly-GR levels in frontal and temporal cortex and age of onset, as well as between levels in occipital cortex (but not in the cerebellum) and age at death and disease duration. Soluble poly-GR levels in the temporal cortex and variably frontal and occipital cortex also showed a trend to negatively correlate with age of onset, age at death and disease duration.

**Table 3:** Correlation of levels of soluble and insoluble poly-GP, poly-GA and poly-GR with parameters of clinical severity (age at death, age of onset, disease duration)

	Poly-GP		Poly-GA		Poly-GR	
	Soluble	Insoluble	Soluble	Insoluble	Soluble	Insoluble
*Age at death*						
FC	− 0.44	− 0.42	− 0.22	− 0.22	− 0.14	− **0.72** *(0.007)*
TC	− 0.48 *(0.10)*	− 0.27	− 0.03	0.02	− 0.51 *(0.08)*	− **0.61** *(0.03)*
OC	− 0.40	− 0.14	0.44	0.27	− 0.53 *(0.07)*	− 0.54 *(0.06)*
Cb	− 0.08	− 0.11	− 0.05	− 0.22	− 0.01	− 0.12
*Age of onset*						
FC	− 0.45	− 0.34	− 0.49 *(0.09)*	− 0.39	− 0.06	− 0.53 *(0.07)*
TC	− **0.60** *(0.03)*	− 0.38	− 0.11	− 0.20	− 0.52 *(0.07)*	− 0.55 *(0.06)*
OC	− 0.48 *(0.10)*	− 0.17	0.26	0.17	− 0.47 *(0.10)*	− 0.45
Cb	− 0.16	− 0.23	0.08	− 0.41	0.15	− 0.12
*Disease duration*						
FC	− 0.19	− 0.50 *(0.09)*	0.35	0.08	− 0.48 *(0.10)*	− **0.66** *(0.02)*
TC	− 0.08	0.08	0.23	0.36	− 0.49 *(0.09)*	− **0.64** *(0.02)*
OC	− 0.12	− 0.06	0.44	0.17	− 0.34	− 0.52 *(0.07)*
Cb	0.21	0.33	− 0.08	0.23	− 0.26	0.12

Spearman’s rank correlation coefficient ρ is shown for correlation of soluble and insoluble poly-GP, poly-GA and poly-GR in frontal cortex (FC), temporal cortex (TC), occipital cortex (OC) and cerebellum (Cb) with age at death, age of onset and disease duration. Correlation coefficients with statistical significance are shown in bold. For all values showing a statistically significant or a trend of correlation, *p*-values are specified between brackets

In order to explore whether the higher poly-GR levels in the homozygous *C9orf72* mutation case were the sole contributor to the significant correlation, these analyses were repeated excluding this case. A negative correlation between insoluble poly-GR levels in the frontal cortex and age at death (ρ = − 0.64, *p* = 0.03) and a trend between insoluble poly-GR levels in the frontal and temporal cortex and disease duration were still present (frontal cortex ρ = − 0.57, *p* = 0.06; temporal cortex ρ = − 0.54, *p* = 0.08) (Additional file 1 Table 4).

**Fig. 5:**
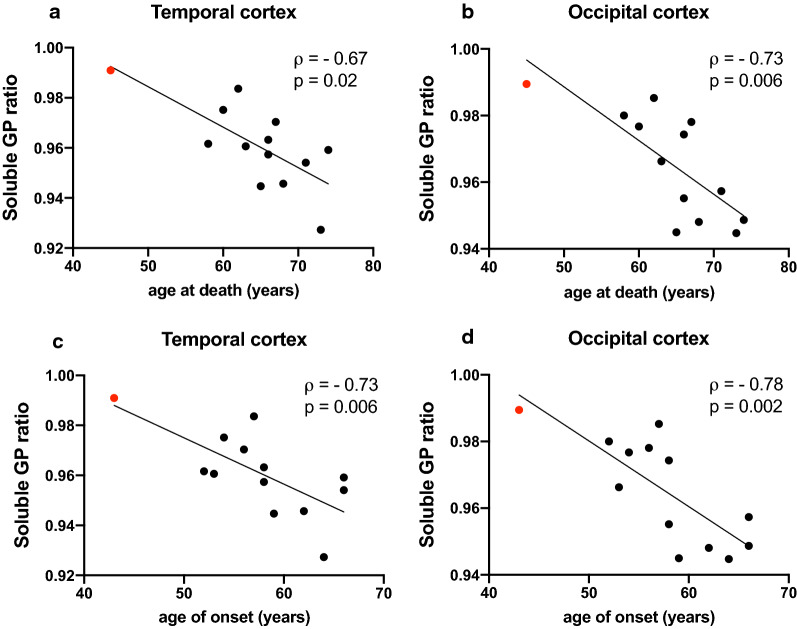
Soluble poly-GP ratios correlate with clinical parameters. Correlation of soluble poly-GP ratio in temporal and occipital cortex with age at death (**a**–**b**) and age of onset (**c**–**d**). The data points in the graphs represent individual *C9orf72* cases (with homozygous *C9orf72* mutation case in red). Spearman’s correlation analysis performed with Spearman’s correlation coefficient ρ. All data are shown in Table 4

In addition to poly-GR levels showing a relationship with clinical severity, soluble poly-GP levels appeared higher in subjects with earlier disease onset in the frontal, temporal and occipital cortex, reaching statistical significance in the temporal cortex (Table 3). As this association was not seen for insoluble poly-GP levels, this raised the possibility of a more specific role for soluble poly-GP in disease pathogenesis. In line with this finding, there was a negative correlation between the poly-GP solubility ratio (soluble over total poly-GP) and age of onset and death in the temporal and occipital cortex (Fig. 5, Table 4). Even though the differences in solubility are small, this implicates that cases with relative higher levels of soluble poly-GP (vs insoluble) show more severe disease. Whilst the correlation of soluble poly-GP levels with clinical parameters was no longer significant once the homozygous *C9orf72* mutant case was excluded, (Additional file 1: Table 5), the correlations between poly-GP solubility ratios and clinical severity parameters remained significant (Additional file 1: Table 6).

**Table 4:** Correlation of levels of solubility ratios of poly-GP, poly-GA and poly-GR with parameters of clinical severity (age at death, age of onset, disease duration)

Ratio soluble DPR/total	Poly-GP	Poly-GA	Poly-GR
*Age at death*			
FC	− 0.35	0.20	0.28
TC	− **0.67** (0.015)	− 0.05	0.15
OC	− **0.73** (0.006)	− 0.21	− 0.23
Cb	− 0.43	0.00	0.25
*Age of onset*			
FC	− 0.61 *(0.05)*	− 0.18	0.32
TC	− **0.73** *(0.006)*	− 0.08	− 0.03
OC	− **0.78** *(0.002)*	− 0.27	− 0.23
Cb	− 0.49 *(0.09)*	0.14	0.40
*Disease duration*			
FC	− 0.09	0.24	− 0.22
TC	− 0.26	0.03	0.05
OC	− 0.36	− 0.07	0.03
Cb	− 0.05	− 0.06	− 0.38

Spearman’s rank correlation coefficient ρ is shown for correlation of soluble ratio (over total) of poly-GP, poly-GA and poly-GR in frontal cortex (FC), temporal cortex (TC), occipital cortex (OC) and cerebellum (Cb) with age at death, age of onset and disease duration. Correlation coefficients with statistical significance are shown in bold. For all values showing a statistically significant or a trend of correlation, *p*-values are specified between brackets

## Discussion

Translation of the hexanucleotide repeat expansion in the *C9orf72* gene into proteins with repeating dipeptides is one of the putative pathomechanisms of C9-FTD disease. Quantitative biochemical data on soluble and insoluble DPR proteins in human C9 disease are however exceedingly sparse. To our knowledge, our study is the second to report on soluble and insoluble fractions of poly-GP and poly-GA [13] and the first one on soluble and insoluble poly-GR in different brain areas of human C9-FTD, providing insights into the solubility, distribution and abundance of the sense dipeptide repeat proteins poly-GA, poly-GP and poly-GR in relation to clinically affected and less affected areas.

### Relative levels and solubility of DPRs across the brain

Our study shows that poly-GA and poly-GP are the more abundant DPR proteins whereas poly-GR levels are much closer to control levels and therefore likely to be much less abundant. This confirms previous immunohistochemical studies reporting on the relative abundance of DPR aggregates [8, 19, 25, 26, 28], however direct comparison between the different DPR proteins warrants caution, as different affinities of their respective antibodies used in the MSD assay cannot be entirely ruled out.

We show that the cerebellum has the highest levels of both soluble and insoluble poly-GP, which is consistent with the MSD data from Gendron et al. [13]. In addition, cerebellum levels of soluble poly-GR and poly-GA are also higher in comparison to those in the frontal cortex. However, in contrast to poly-GP, insoluble poly-GR and poly-GA show no significantly different distribution across several cortical areas and cerebellum, a finding in line with the distribution pattern observed in immunohistochemical studies [8, 9, 18–20, 22, 23, 28]. This indicates that poly-GA and poly-GR are differentially soluble in different brain regions. It will be intriguing to determine why this is the case, as it could shed light on selective vulnerability of different brain regions to DPRs. Potential mechanisms include differential chaperone function or degradation pathways between different brain regions. Why differential solubility across vulnerable and less vulnerable regions was observed for poly-GR and poly-GA, but not poly-GP, is not clear, but may be due to the different biochemical properties of each DPR. A recent study suggests that chimeric DPR species containing both poly-GA and poly-GP may be produced in C9-FTD/ALS patient brains [21]. Our data shows that poly-GP and poly-GA have differential solubility profiles across brain regions, in addition to very different solubility profiles overall (poly-GP is largely soluble and poly-GA is largely insoluble, as also reported by Gendron et al. [13]). This indicates that, while still consistent with the presence of chimeric DPRs, the biochemical properties of a meaningful proportion of the DPRs present in patient brain are driven by a single DPR species.

The cerebellum not only stands out due to a high DPR load, but also because of a substantially higher relative DPR solubility (i.e. proportion of soluble DPR over total DPR). In particular poly-GA shows a striking change in solubility profile in the cerebellum: whereas poly-GA is known to be aggregation-prone [31] and also in our study largely exists in aggregated insoluble form, in the cerebellum the majority of protein is present in its soluble form. A different, less aggregation-prone, biophysical environment, particular to the cerebellum, could be a potential explanation for a ratio favouring soluble over insoluble DPRs.

### The correlation between DPRs, neurodegeneration and clinical parameters

Our data show that soluble DPRs are less abundant in brain regions that are more affected by neurodegeneration in C9-FTD (i.e. temporal and frontal cortex) and are present at higher levels in the cerebellum, an area usually considered to be less involved. It is possible that the affected brain regions in post-mortem brain tissue show less soluble DPR levels due to end-stage neuronal loss, consistent with the observation of decreasing number of DPR-positive cells with increasing age and neurodegeneration in poly-PR overexpressing mice [34]. This might implicate that soluble DPRs play an active role in causing neurodegeneration in earlier disease stages or alternatively do not play any causative role at all. Given the difficulties in interpreting end-stage post-mortem data, further functional studies will be required to pick apart these possibilities. For instance, mouse model studies have revealed that poly-GA aggregation is necessary for toxicity [33] whereas poly-GR appears to mediate neurotoxicity both in diffuse and aggregated forms [32]. The finding that, in contrast to the soluble forms, insoluble poly-GA and poly-GR levels are not different across the different areas may suggest that aggregation confers some neuroprotective properties. The high levels of soluble DPRs in the cerebellum might fit with the explanation of selective neuronal vulnerability. However, alternatively, given a previous report on a correlation between cerebellar poly-GP inclusions and the development of cognitive symptoms in a C9-ALS cohort [13], and cerebellar changes observed in C9-FTD patients using MRI [5] a more active role for DPRs in the cerebellum needs to be considered as well.

Our study highlights a negative correlation between poly-GR levels and clinical parameters of severity (age at death, age of onset and disease duration). The association reaches statistical significance for insoluble poly-GR in frontal and temporal cortex with age at death and disease duration only, but a trend is noted for both soluble and insoluble poly-GR with all clinical parameters across all brain regions excluding cerebellum. Evidence of a neurotoxic role for poly-GR in animal and cellular *C9orf72* models is extensive [3, 30], still the role of poly-GR in human disease remains controversial. Mackenzie et al. did not find a correlation between poly-GR inclusion density in the frontal cortex and neurodegeneration, age at onset or disease duration [19]. Yet, two recent studies point to an association with neurodegeneration with one study showing a correlation between poly-GR inclusions and degree of neurodegeneration in the frontal cortex [26] and the other showing more abundant poly-GR inclusions in clinically affected areas (excluding cerebellum from their analysis) [25].

Some of the differences between our findings and these earlier studies might be explained by two crucial differences in study methodology. First of all, our study relies on biochemical measurements of soluble and insoluble DPRs. It is very likely that measurement of insoluble fractions through MSD immunoassays show slightly different results than quantification of aggregated DPR inclusions by means of immunohistochemistry, an observation which has already been made for other proteins [7]. Another factor probably contributing to diverging results in between studies would be patient selection: whereas the two larger previous studies relied on a mix of clinicopathological phenotypes (i.e. FTLD, FTLD-MND and MND only) [19, 26] and the smaller study of Saberi et al. consisted of 5 MND patients, the vast majority of the cases in our study represented FTLD without MND. As previous work has demonstrated substantial differences in poly-GR density and distribution across the different clinico-pathological subtypes [26], it is not entirely surprising that depending on the phenotypes included different results are obtained. An additional role for poly-GR was recently suggested by a study reporting a positive correlation between symmetrically dimethylated poly-GR (GR-SDMA) inclusions and age of onset and age at death [15]. This difference between GR-SDMA inclusions and poly-GR measured by MSD may be due to GR-SDMA being a minority of the total poly-GR and highlights that methylation of poly-GR is likely to alter its function; and in a potentially protective manner.

In contrast to poly-GR, poly-GA did not show any consistent relationships with clinical parameters. Mackenzie et al. [19] report on a weak correlation between poly-GA levels and age of onset in the frontal cortex and notably our data reveal a similar trend. Finally, soluble poly-GP in the temporal cortex showed a significant negative correlation with age of onset and soluble poly-GP ratios (soluble over total poly-GP levels) negatively correlate with age of onset and death in the temporal and occipital cortex. The latter correlations remained significant after excluding the homozygous case from the analysis. It is interesting that this correlation persists even though the actual differences in solubility ratios are very small. Further work is needed to assess the relevance of such small changes to poly-GP solubility. Yet, these findings raise the possibility that soluble poly-GP might play a role in disease pathogenesis. Notably, a recent paper on SCA36 showed soluble poly-GP levels in areas affected by neurodegeneration, suggestive of a potential neurotoxic role of soluble poly-GP [21].

One of the limitations of our study is a relatively small sample size and therefore our findings will require further validation. Furthermore, some of the correlations with clinical parameters are at least partially determined by the DPR values in the homozygous C9 case, an issue that would be resolved with a larger sample size. As we have previously shown that the homozygous case has lower levels of *C9orf72* repeat transcripts [11] this raises the possibility that other parameters may also be relevant such as C9orf72 levels. Indeed, there are several areas beyond the scope of this exploratory study, which might be interesting directions for further research such as any potential associations with histopathologic quantification of DPR inclusions, amount of TDP-43 pathology, RNA foci, *C9orf72* repeat length, *C9orf72* expression and methylation levels.

## Conclusion

Our study is the first biochemical quantification of all soluble and insoluble sense DPR levels across affected and less affected areas in C9-FTD brains. The observation that poly-GR levels are associated with clinical parameters of severity raises the possibility that poly-GR not only in disease models but also in human disease has a neurotoxic role. Our study also points to a possible link between poly-GP solubility and clinical severity and shows lower soluble DPR levels in clinically affected areas which might suggest a role for soluble DPRs in early disease stages, or, alternatively, could mean they do not play a major role in disease. This will need further studies by in vitro and in vivo experimental studies in *C9orf72* cell culture and animal models.

## Supplementary information

**Additional file 1.** Supplementary data.

## Data Availability

All data generated or analysed during this study are included in this published article.

## Abbreviations

FTD: Frontotemporal degeneration
FTLD: Frontotemporal lobar degeneration
DPR: Dipeptide repeat protein
MSD: Meso Scale Discovery
SCA36: Spinocerebellar Ataxia 36
