# Supplementary material for: Soluble and insoluble dipeptide repeat protein measurements in C9orf72-frontotemporal dementia brains show regional differential solubility and correlation of poly-GR with clinical severity
Source: Acta Neuropathol Commun. 2020 Nov 9;8:184. doi: 10.1186/s40478-020-01036-y (PMC7650212; doi:10.1186/s40478-020-01036-y)
Supplement: Supplementary file 1 — Additional file 1. Supplementary data. [file 40478_2020_1036_MOESM1_ESM.docx]

Soluble and insoluble dipeptide repeat protein measurements in *C9orf72*-frontotemporal dementia brains show regional differential solubility and correlation of poly-GR with clinical severity

Annelies Quaegebeur, Idoia Glaria, Tammaryn Lashley and Adrian M. Isaacs

**Supplementary data**

**Supplementary tables**

**Additional file 1: table 1**. Demographical details of neurologically healthy control subjects.

| Number | Gender | Age at death | Postmortem  interval (hr:min) | Cause of death |
| --- | --- | --- | --- | --- |
| 14 | M | 76 | 79:00 | Multi-organ failure |
| 15 | M | 84 | 79:10 | Metastatic prostate carcinoma |
| 16 | F | 82 | 99:00 | Bronchopneumonia |
| 17 | F | 82 | 91:20 | Not available |
| 18 | F | 80 | 49:10 | Pancreatic carcinoma |

**Additional file 1: table 2.** DPR levels in the homozygous C9orf72-FTD brain versus the mean of the non-homozygous C9-FTD subjects.

|  | | FC | TC | OC | Cb |
| --- | --- | --- | --- | --- | --- |
| GP soluble | Non-homozygous | 1035.2 ±  193.6 | 2478.3 ±  449.8 | 2741.9 ±  394.9 | 34910.8 ±  10200.0 |
|  | Homozygous | 8881.1 | 15501.4 | 12940.2 | 19416.1 |
| GP insoluble | Non-homozygous | 44.5 ± 5.8 | 92.8 ± 16.0 | 90.6 ± 13.0 | 126.2 ± 22.1 |
|  | Homozygous | 148.8 | 140.9 | 137.4 | 84.4 |
| GA soluble | Non-homozygous | 18987.8 ±  3109.1 | 20499.2 ±  1973.1 | 26265.0 ±  2913.8 | 326366.3 ±  172834.3 |
|  | Homozygous | 24744.3 | 10828.8 | 13537.8 | 2418.0 |
| GA insoluble | Non-homozygous | 70134.9 ±  10316.14 | 88766.8 ±  9872.0 | 79502.0 ±  21338.4 | 64453.58 ±  11129.6 |
|  | Homozygous | 197605.0 | 125483.0 | 74756.5 | 77814.9 |
| GR soluble | Non-homozygous | 7.7 ± 2.8 | 26.0 ± 6.8 | 48.5 ± 14.1 | 40.1 ± 7.5 |
|  | Homozygous | 71.7 | 113.7 | 89.5 | 33.9 |
| GR insoluble | Non-homozygous | 25.1 ± 5.6 | 29.3 ± 5.1 | 29.8 ± 3.9 | 40.2 ± 9.5 |
|  | Homozygous | 121.2 | 143.6 | 94.2 | 35.1 |

DPR concentration values measured by MSD of the homozygous C9orf72-FTD case *versus* mean of the remaining, non-homozygous, cases are shown for frontal (FC), temporal (TC), occipital (OC) cortex and cerebellum (Cb), for both soluble and insoluble fractions. Data are represented as mean ± SEM.

**Additional file 1: table 3.** Correlation analysis of levels of soluble and insoluble DPRs with *post-mortem* interval.

|  | Poly-GP | | Poly-GA | | Poly-GR | |
| --- | --- | --- | --- | --- | --- | --- |
|  | Soluble | Insoluble | Soluble | Insoluble | Soluble | Insoluble |
| FC | 0.25 | -0.18 | 0.36 | 0.48 | -0.45 | -0.13 |
| TC | 0.24 | 0.40 | 0.41 | 0.50 | -0.15 | -0.27 |
| OC | 0.37 | 0.52 | 0.72 *(0.01)* | 0.77 *(0.01)* | -0.42 | -0.39 |
| Cb | -0.03 | -0.06 | -0.34 | 0.35 | -0.54 | -0.18 |

Spearman’s rank correlation coefficient ρ for soluble and insoluble concentrations of poly-GP, poly-GA and poly-GR in frontal cortex (FC), temporal cortex (TC), occipital cortex (OC) and cerebellum (Cb). All tests show non-significant correlation apart from soluble and insoluble poly-GA levels in the occipital cortex showing a positive correlation with the postmortem interval with p-values indicated between brackets. As the positive correlation implies higher poly-GA levels on longer postmortem interval this result is considered unlikely to be relevant.

**Additional file 1: table 4.** Correlation analysis between levels of soluble and insoluble poly-GR and clinical parameters with and without homozygous *C9orf72* mutation case.

**Poly-GR (including homozygous case)**

|  | Age at death | | Age of onset | | Disease duration | |
| --- | --- | --- | --- | --- | --- | --- |
| Poly-GR | Soluble | Insoluble | Soluble | Insoluble | Soluble | Insoluble |
| FC | -0.14 | **-0.72** *(0.007)* | -0.06 | -0.53 *(0.07)* | -0.48 *(0.10)* | **-0.66** *(0.02)* |
| TC | -0.51 *(0.08)* | **-0.61** *(0.03)* | **-**0.52 *(0.07)* | -0.55 *(0.06)* | -0.49 *(0.09)* | **-0.64** *(0.02)* |
| OC | -0.53 *(0.07)* | -0.54 *(0.06)* | -0.47 *(0.10)* | -0.45 | -0.34 | -0.52 *(0.07)* |
| Cb | -0.01 | -0.12 | 0.15 | -0.12 | -0.26 | 0.12 |

**Poly-GR (excluding homozygous case)**

|  | Age at death | | Age of onset | | Disease duration | |
| --- | --- | --- | --- | --- | --- | --- |
| Poly-GR | Soluble | Insoluble | Soluble | Insoluble | Soluble | Insoluble |
| FC | 0.12 | **-0.64** *(0.03)* | 0.23 | -0.40 | -0.32 | -0.57 *(0.06)* |
| TC | -0.37 | -0.50 *(0.10)* | -0.39 | -0.42 | -0.35 | -0.54 *(0.08)* |
| OC | -0.44 | -0.41 | -0.39 | -0.30 | -0.23 | -0.38 |
| Cb | -0.03 | -0.10 | 0.13 | -0.10 | -0.27 | 0.18 |

Spearman’s rank correlation coefficient ρ is shown for correlation of soluble and insoluble poly-GR in frontal cortex (FC), temporal cortex (TC), occipital cortex (OC) and cerebellum (Cb) with age at death, age of onset and disease duration. Correlation coefficients with statistical significance are shown in bold. For all values showing a statistically significant or a trend of correlation, p-values are specified between brackets.

**Additional file 1: table 5.** Correlation analysis between soluble poly-GP levels and clinical parameters with and without homozygous *C9orf72* mutation case.

**Poly-GP (including homozygous case)**

|  | Age at death | | Age of onset | | Disease duration | |
| --- | --- | --- | --- | --- | --- | --- |
| Poly-GP | Soluble | Insoluble | Soluble | Insoluble | Soluble | Insoluble |
| FC | -0.44 | -0.42 | -0.45 | -0.34 | -0.19 | -0.50 *(0.09)* |
| TC | -0.48 *(0.10)* | -0.27 | **-0.60** *(0.03)* | -0.38 | -0.08 | 0.08 |
| OC | -0.40 | -0.14 | -0.48 *(0.10)* | -0.17 | -0.12 | -0.06 |
| Cb | -0.08 | -0.11 | -0.16 | -0.23 | 0.21 | 0.33 |

**Poly-GP (excluding homozygous case)**

|  | Age at death | | Age of onset | | Disease duration | |
| --- | --- | --- | --- | --- | --- | --- |
| Poly-GP | Soluble | Insoluble | Soluble | Insoluble | Soluble | Insoluble |
| FC | -0.28 | -0.26 | -0.30 | -0.16 | 0.04 | -0.36 |
| TC | -0.34 | -0.19 | -0.48 *(0.11)* | -0.31 | 0.17 | 0.25 |
| OC | -0.24 | -0.02 | -0.30 | -0.34 | 0.13 | 0.15 |
| Cb | -0.09 | -0.09 | -0.19 | -0.25 | 0.24 | 0.42 |

Spearman’s rank correlation coefficient ρ is shown for correlation of soluble and insoluble poly-GP in frontal cortex (FC), temporal cortex (TC), occipital cortex (OC) and cerebellum (Cb) with age at death, age of onset and disease duration. Correlation coefficients with statistical significance are shown in bold. For all values showing a statistically significant or a trend of correlation, p-values are specified between brackets.

**Additional file 1: table 6**. Correlation analysis between soluble poly-GP ratio and clinical parameters with and without homozygous *C9orf72* mutation case.

| Soluble poly-GP | Including homozygous case | | Excluding homozygous case | |
| --- | --- | --- | --- | --- |
| **Ratio** | Age at death | Age of onset | Age at death | Age of onset |
| FC | -0.35 | -0.61 *(0.05)* | -0.14 | -0.48 |
| TC | **-0.67** *(0.015)* | **-0.73** *(0.006)* | -0.57 *(0.05)* | **-0.65** *(0.025)* |
| OC | **-0.73** *(0.006)* | **-0.78** *(0.002)* | **-0.65** *(0.024)* | **-0.72** *(0.011)* |
| Cb | -0.43 | -0.49 *(0.09)* | -0.50 | -0.55 *(0.07)* |

Spearman’s rank correlation coefficient ρ is shown for correlation of soluble poly-GP ratio in frontal cortex (FC), temporal cortex (TC), occipital cortex (OC) and cerebellum (Cb) with age at death and age of onset. Correlation coefficients with statistical significance are shown in bold. For all values showing a statistically significant or a trend of correlation, p-values are specified between brackets.

**Supplementary figures**

**Additional file 1: Figure 1. Validation of MSD immunoassays.** Specificity of poly-GR, poly-GP and poly-GA immunoassays was validated with a cross-reactivity assay (a-c) loading lysates from HeLa cells (45 µg per well) transfected to express different C9orf72 RAN translated proteins: (GR)100, (GA)100, (PR)100, (GGGGCC)92 plasmids and non-transfected cells. Counts measured for each condition are shown, from which the background value obtained for the blank diluent is subtracted (n=1).

**Additional file 1: Figure 2. Relative soluble and insoluble DPR values *versus* healthy controls.** Relative DPR values in the C9orf72-FTD subjects (C9) as measured by MSD in comparison to the values measured in the healthy control subjects (ctrl) in frontal, temporal, occipital cortex and cerebellum. Data are shown as mean ± SEM. Statistical analyses are shown in Table 2.

**Additional file 1: Figure 3: DPR protein solubility is DPR- and region-specific**. The ratio of soluble (S) and insoluble (INS) DPR protein concentrations over total DPR protein levels were calculated across different brain regions. (a-d) Ratio of soluble and insoluble poly-GP; (e-h) ratio of soluble and insoluble poly-GA; (i-l) ratio of soluble and insoluble poly-GR. All data are mean ± SEM. **p< 0.01, ***p< 0.001, ****p< 0.0001.
